# Preliminary Effectiveness of mHealth App-Based Pelvic Floor Muscle Training among Pregnant Women to Improve Their Exercise Adherence: A Pilot Randomised Control Trial

**DOI:** 10.3390/ijerph19042332

**Published:** 2022-02-18

**Authors:** Aida Jaffar, Sherina Mohd Sidik, Chai Nien Foo, Noor Azimah Muhammad, Rosliza Abdul Manaf, Nazhatussima Suhaili

**Affiliations:** 1 Department of Psychiatry, Faculty of Medicine and Health Sciences, Universiti Putra Malaysia, Serdang 43400, Malaysia; aida@upnm.edu.my; 2 Primary Care Unit, Faculty of Medicine and Defence Health, Universiti Pertahanan Nasional Malaysia, Kuala Lumpur 57000, Malaysia; 3 Department of Population Medicine, Faculty of Medicine and Health Sciences, Universiti Tunku Abdul Rahman, Kajang 43000, Malaysia; 4 Department of Family Medicine, Faculty of Medicine, Universiti Kebangsaan Malaysia, Kuala Lumpur 56000, Malaysia; drazimah@ppukm.ukm.edu.my; 5 Department of Community Health, Faculty of Medicine and Health Sciences, Universiti Putra Malaysia, Serdang 43400, Malaysia; rosliza_abmanaf@upm.edu.my; 6 Klinik Kesihatan Ampang, Ministry of Health, Ampang 68000, Malaysia; drshima.suhaili@gmail.com

**Keywords:** mHealth app, mobile application, pelvic floor muscle training, urinary incontinence, pregnancy, maternal health, randomised control trial, pilot feasibility study

## Abstract

This pilot randomised control trial (RCT) aimed to evaluate the feasibility and preliminary effectiveness of conducting a full-powered trial for a newly developed pelvic floor muscle training (PFMT) app among pregnant women with urinary incontinence (UI) in Malaysia. This was a prospective, single-centre, single-blind, parallel, randomised controlled, pilot feasibility study—the Kegel Exercise Pregnancy Training app (KEPT app) trial. In total, 26 pregnant women with urinary incontinence from an urban healthcare clinic were recruited and randomly assigned to either intervention or waitlist control group. The intervention group received the KEPT app, while the control group received usual antenatal care (waitlist control). Of the 26 pregnant women, 16 (61.5%) completed the two-month follow-up. The recruitment rate was 54.2%, and the retention rate was 62.5% in the intervention group and 60% in the control group. There was a significant difference between intervention and control groups’ baseline measurement in the severity of UI (*p* = 0.031). The app improved their knowledge (*p* = 0.011) and self-efficacy (*p* = 0.038) after the first month and attitude (*p* = 0.034) after two months of intervention, compared with the control group. This study supports the feasibility of our future cluster RCT. The KEPT app demonstrates a promising effect in improving PFMT attitude and self-efficacy and potentially enhancing exercise adherence among pregnant women with UI. Trial registration: This study was prospectively registered on ClinicalTrials.gov on 19 February 2021 (NCT04762433).

## 1. Introduction

Pelvic floor muscle training (PFMT), or Kegel exercise, is the gold standard and is recommended for pregnant women to strengthen pelvic floor muscles [1,2]. The correct performance of PFMT may help first-time pregnant women to shorten their first and second stages of labour [3]. The same exercise can prevent pelvic floor dysfunction, for example, urinary incontinence, which commonly occurs in late pregnancy and the early post-partum period [4]. Moreover, a meta-analysis study has demonstrated positive results with training exercise among pregnant women at any parity in improving quality of life [5].

Urinary incontinence (UI) is defined as involuntary urinary leakage involving about two-fifth of our local population in a single-centred, cross-sectional study [6,7]. Worldwide UI prevalence demonstrated variations ranging from 9% to 75% [8]. Having UI does not add risk to maternal mortality but affects their quality of life and causes psychological morbidities [9,10]. Additionally, they may suffer difficulties in social–emotional relationships, performing exercises, restriction travelling, and sleeping disturbances [11].

Previous studies highlighted that pregnant women face challenges adhering to PFMT, as they consider having UI is ‘normal’. This misconception led to a barrier in seeking help from healthcare providers. Furthermore, there were limited credible sources for PFMT information, [12] preventing them from knowing the benefit of the exercise during pregnancy. Recent guidelines proposed that three sets of exercises are needed to improve the pelvic floor muscle strength [12]. Three sets of daily exercises during their busy schedules make them experience difficulties remembering to perform the exercise [13]. Subsequently, health personnel struggles to discuss and offer pelvic exercise advice due to inadequate knowledge [14], which is not routinely practised [15]. These factors will further reduce the availability of the services and affect the accessibility of PFMT to pregnant women [16].

mHealth apps have shown their effectiveness in self-management pregnancy and improving healthcare delivery [17]. Evidence suggests that the apps can provide audio guidance for PFMT [18] and reminders to improve motivation and adherence [18]. Furthermore, delivering pregnancy-related education and self-management using the mHealth app reported promising outcomes [17] and may be used for self-empowering among pregnant women.

Therefore, this pilot RCT aimed to assess the preliminary effectiveness of a newly developed, validated mHealth app—Kegel Exercise Pregnancy Trial (KEPT app) [19]. Their knowledge, attitude, practice, self-efficacy, and adherence to PFMT with their severity urinary incontinence symptoms and quality of life were assessed in this pilot RCT.

## 2. Materials and Methods

### 2.1. Design Overview

An eight-week, two-arm, parallel-group, pilot RCT was undertaken at an urban government health clinic in Ampang, Selangor. Participants were randomly allocated to the intervention group who received the Kegel Exercise Pregnancy Training app (KEPT app) or waitlist control (receiving KEPT app after completing the study). The assessments were conducted at baseline, one month, and after two months of the study. The study was prospectively registered with ClinicalTrials.gov on 19 February 2021 (NCT04762433). The study protocol was designed and reported according to the Consolidated Standards of Reporting Trials (CONSORT) extension for randomised pilot and feasibility trials [20,21] and has been published recently [22].

### 2.2. Participants

Women aged 18 and above with urinary incontinence were recruited from June 2021 to September 2021. Detailed inclusion and exclusion criteria are described in Table 1. Participants were recruited using e-poster strategies delivered via WhatsApp by the researcher’s team. The pilot RCT obtained ethics approval from the Ethics Committee for Research Involving Human Subjects, Universiti Putra Malaysia (JKEUPM-2019-368) Medical Research and Ethics Committee (MREC), Ministry of Health Malaysia (NMRR-19-412-45606) in August 2019.

The trial was undertaken in compliance with the Declaration of Helsinki [23]. Interested participants were provided with an online participant information statement from the researcher’s team. They filled in the google forms survey for our team to assess their study eligibility. An online consent form was provided to eligible participants for their digital signature prior to the study commencement. The study protocol is designed and has been published elsewhere [22].

### 2.3. Intervention

Participants allocated to the intervention group were provided 8-weeks behavioural change intervention (pelvic floor muscle training) via a newly developed mHealth app (KEPT app). The KEPT app was an interactive Android version that focused on the PFMT program (educational video, training timer, UI symptoms calendar chart, daily reminder notification, progress chart, and frequently asked questions). KEPT app recommendations were promoted through an evidence-based program that has been validated [18] and undergone expert usability testing [26]. The following program components were featured:

- Educational video: Participants watched a PFMT educational video demonstrated by a certified physiotherapist for six minutes. The video has been approved for education in the rehabilitation department tertiary hospital.
- Training timer: Participants performed the exercise according to the tailored timer performance ability (beginner: 2 s contraction, intermediate: 6 s contraction, and expert: 10 s contraction) and 6 s rest between each repetition, every day for three times daily. There were slow-velocity, close-to-maximum contractions of exercise. The first (beginner) performed the quick muscle contractions for two seconds and rested for six seconds while breathing normally. The quick contractions were required to perform 3 times daily, for 10 repetitions each cycle. After gaining confidence and skills, they proceeded to the longer durations, where the same muscles they contracted with longer durations of 6 to 10 seconds, for 10 repetitions, 3 times daily.
- Symptoms calendar charting: Participants recorded their UI symptoms for their self-monitoring.
- Progress chart: Participants could self-monitor their progress of UI symptoms and PFMT adherence.
- Frequently asked questions: Participants could read further the details of anatomy and PFMT techniques.
- Notification reminder: Participants received a daily notification to remind their pelvic exercise.

### 2.4. Control Group

Participants allocated to the control group were provided with the KEPT app after completing the eight-week follow-up appointment. They continued their usual antenatal follow-up as scheduled.

### 2.5. Outcome Measures (Preliminary Effectiveness)

The feasibility of this study was assessed by determining the proportions of respondents who meet the eligibility criteria, recruitment rate, and retention rate [27]. All outcomes were measured at baseline, one month, and two months post-intervention. Participant completion of each outcome measure was traced to measure the feasibility of the data collection procedures [28]. The primary outcome was to assess the PFMT adherence among the study participants. Secondary outcomes, including urinary incontinence, quality of life, PFMT knowledge, attitude, practice, and self-efficacy, are described in Table 2.

### 2.6. Sample Size

It is not required to have a powered sample size for the pilot study [32]. Previous analysis suggested a minimum of 12–30 participants per group as an appropriate sample in feasibility studies [33] and pilot studies [34]. This study was anticipated to have 64 participants within 2 months duration.

### 2.7. Randomisation and Blinding

A randomisation app (RRApp) generated the randomisation sequence. Participants stratification by primigravida and multigravida to minimise the selection bias. A concealed envelope was provided to a non-researcher to reveal the assigned intervention and control group. This study was a single-blinded study, in which the researchers involved were blinded to participant group allocation [35], as it was not feasible and possible to blind the participant due to the nature of the intervention and control conditions.

### 2.8. Statistical Methods

All analyses were performed utilising the Statistical Package for the Social Sciences version 27.0 (IBM, New York, NY, USA) [36,37]. Data are presented according to normality testing (Shapiro–Wilk test) distribution with mean and standard deviation (SD) or median, interquartile range (IQR) for continuous variables, and counts (percentages) for categorical variables. Baseline characteristics of the participants and the study outcomes were determined using either *t*-test or Mann–Whitney U test accordingly between two groups.

Analyses for the preliminary effectiveness outcomes were conducted on an intention-to-treat without computation. The generalised estimating equation (GEE) model was employed to manage the missing values in repeated-measure data. GEE has its robust ability to analyse the intervention effect and interaction effect between time and intervention without replacing the missing data [38]. All analyses with a *p*-value < 0.05 were considered statistically significant.

## 3. Results

### 3.1. Participant Characteristics

A total of 26 pregnant women were randomly allocated to the KEPT app group (*n* = 16) or control group (*n* = 10), aged 21–39 years. The majority were from lower-income status (92.3%, *n* = 24/26), with a minority being primigravida (34.6%, *n* = 9/26), and only one participant had a caesarean section. Less than 20% had provided with PFMT information (14.7%, *n* = 17/26). There were no statistically significant differences between the two groups in baseline characteristics (Table 3) and baseline outcome measures (Table 4), except UI symptoms severity with *p* = 0.031. This finding could be due to the imbalance of the intervention group with Stress UI more than Urge UI, whereby the control group has a similar ratio of Stress UI and Urge UI. The intervention group has significantly less severe in its UI symptoms when compared with the control may attenuate the effectiveness of the intervention due to milder severity of the UI.

### 3.2. Feasibility of the Study

**Figure 1:**
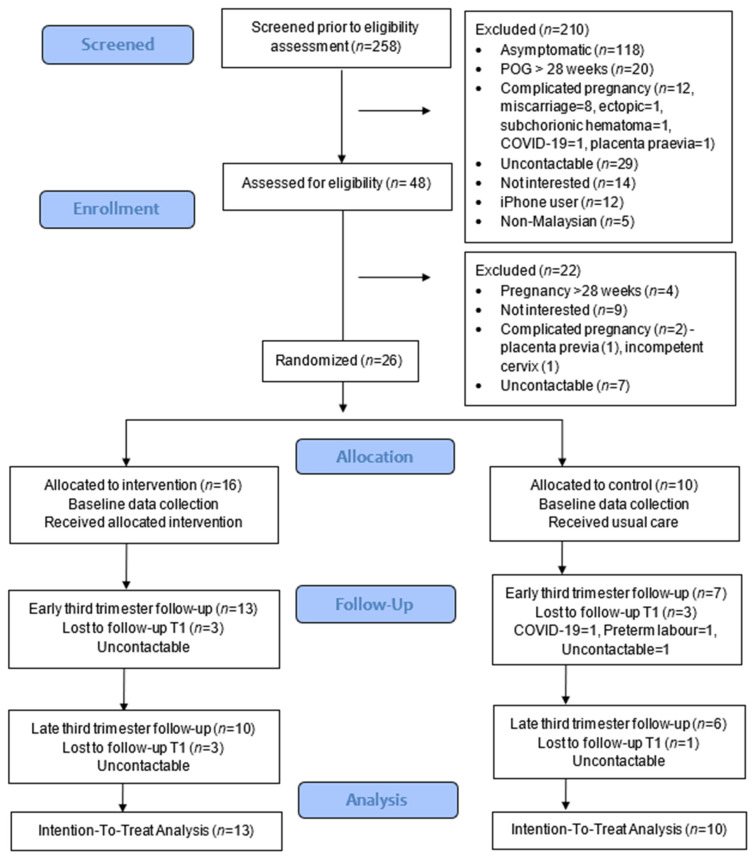
CONSORT study flowchart.

The proportions of respondents who met the eligibility criteria were only 18.6%, with almost half being asymptomatic (45.7%) (Figure 1). The recruitment rate was 54.2%, and the retention rates at 1 month were 81.3% (13/16) for intervention and 70% (7/10) for the control group. At the end of the study, the retention rates were reduced to 62.5% (10/16) for intervention and 60% (6/10) for the control group, and no adverse events were reported in the intervention group. Data collection was feasible using the app and Google Forms.

### 3.3. Primary Outcome

**Figure 2:**
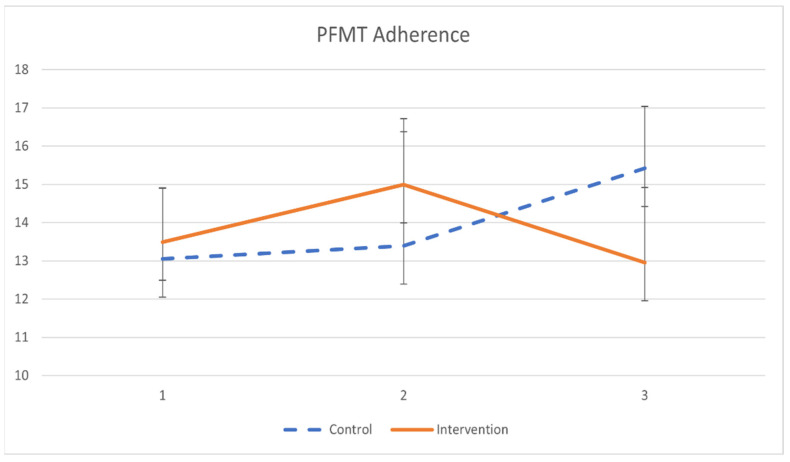
The mean values and standard errors for PFMT adherence of the two groups across the study.

The primary outcomes of pelvic floor muscle training adherence are demonstrated in Table 5 and Figure 2. Participants in the intervention group had minimal significant improvement in PFMT adherence after a 2-month training (β = 0.033, *p* = 0.019). However, the difference in PFMT adherence between groups was not statistically significant.

### 3.4. Secondary Outcomes

#### 3.4.1. PFMT Knowledge, Attitude, Practice, and Self-Efficacy

**Figure 3:**
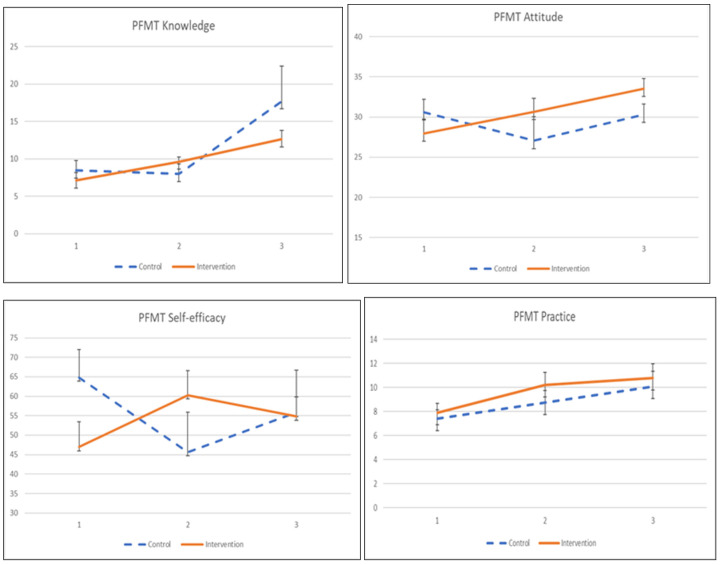
The mean values and standard errors for PFMT knowledge, attitude, practice, and self-efficacy of the two groups across this pilot RCT study.

The adherence to the exercise was influenced by its knowledge, attitude, practice, and self-efficacy, as shown in Table 6 and Figure 3. The interaction effects between intervention and time on knowledge, attitude, and self-efficacy were significant. Participants in the KEPT app group indicated significant knowledge and self-efficacy improvement after a 1-month training (β = 2.968, *p* = 0.011), and β = 6.246, *p* = 0.038). Meanwhile, the participants demonstrated a significantly improved PFMT attitude at 2 months post-intervention, compared with the control group (β = 5.884, *p* < 0.034). PFMT practice was found significant only when compared within the KEPT app group at 2 months (β = 2.668, *p* = 0.018). The differences in PFMT practice between groups were not statistically significant.

#### 3.4.2. Urinary Incontinence and Quality of Life

**Figure 4:**
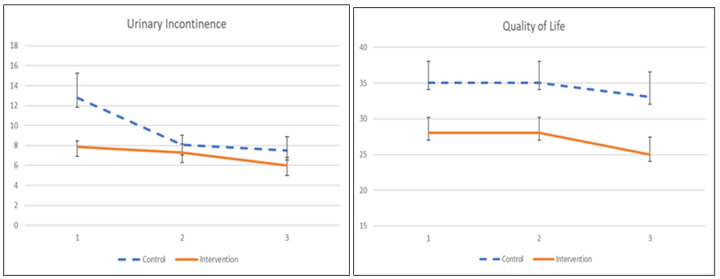
The mean values and standard errors for urinary incontinence severity and quality of life of the two groups across this pilot RCT study.

The UI symptom severity and its quality of life to pelvic floor muscle training are demonstrated in Table 7 and Figure 4. Participants receiving the app showed significant improvement in symptom severity after a 1-month training (β = −4.748, *p* = 0.049) but did not show persistence after 2 months of intervention, as there was no significant treatment effect. The quality of life among participants did not demonstrate any significant improvement at 1 month or 2 months post-intervention.

## 4. Discussion

This study was a single-blind, single-centre, pilot feasibility RCT to assist the feasibility of the proposed future full-size RCT and replicating its miniature [27]. This pilot trial was able to identify the potential challenges of recruitment from one single centre, such as poor recruitment and retention rates. The enabling factors to encourage participation were the excellent relationship with the healthcare centre. Their willingness to assist in disseminating the study information was crucial, leading to the participation of the study respondents.

Self-efficacy has been documented to have a modifier effect on the PFMT, whereby women need to perform the exercise independently [39,40]. Pregnant women must understand the anatomical part of pelvic floor muscle and its function and internalise the ability or physical skills to contract the muscles correctly. This app provided them with an educational video from the physiotherapist and notes to further inform them about the exercise. The app improved their self-efficacy (*p* = 0.038) and knowledge (*p* = 0.011), compared with the control group in the first month. Hence, this study supports the feasibility of the future RCT conducted in 5 months duration until 2 months post-partum.

Effectiveness was reported with minimum improvement in its adherence and the improvement of the symptoms. The knowledge, attitude, practice, and self-efficacy improved in the intervention group. However, a careful interpretation is crucial, as the findings from this study reveal a risk of type II error due to the study’s small sample size and medium effect size [41].

The various unpredictable restrictions in collecting data due to the COVID-19 pandemic added challenges to this study. This study had applied using e-poster information promoted by the assistance from the healthcare providers in the healthcare centre and extended the duration of the recruitment into three months. A low recruitment rate (54.2%) was expected during the COVID-19 pandemic. This study recruited higher than a previous study using social media, with 20–40% [42]. Few suggested strategies, such as including only the highly motivated and committed participants, may be added to our future full RCT eligibility criteria, to improve the recruitment rate and reduce attrition rates.

Despite an acceptable recruitment rate, this study reported less retention, compared with a recent review from sixteen pilot studies [43]. Aside from pandemic-related issues, other factors such as restriction movement order or perhaps psychological stress and financial stress [44] during the pregnancy may have influenced retention. Therefore, significant changes need to be considered, such as adding incentives for both groups with higher amounts in the control group [45] and screening for psychological distress to assist them.

This study was unable to demonstrate improvement in adherence despite improving the self-efficacy towards PFMT. In contrast, the study using audio-app-based PFMT for 6 months in duration among primigravida demonstrated improved adherence with self-efficacy [46]. The result could be because our pilot study was conducted shorter (2 months) than other studies. Women who have undergone supervised training at or over 8 weeks with weekly appointments adhere more effectively than those with unsupervised training [47]. This could be explained unsupervised training might need external motivations to reinforce engagement. The idea of making exercise a more enjoyable experience, aiming to score points and compete with each other, could modify health outcomes behaviour.

PFMT adherence is crucial to improving muscle strength, increasing urethral closure pressure [48], and shortening the muscle length [49] after repeatedly contracting. This study used a validated questionnaire assessing home-based exercise, which was not explicitly designed for PFMT. Using a validated or adapted PFMT adherence questionnaire [50] may result differently from this study. Therefore, a new adapted and validated adherence questionnaire will be used in future RCT.

This study did not report significant findings in the UI symptoms improvement, similar to another 3-month, home-based PFMT intervention among post-partum study participants [51]. Despite no significant improvement, the urinary symptoms were not worsening, with a significant difference in each group. Further qualitative follow-up study may clarify its clinical significance despite being statistically insignificant from the participants’ perspective.

To our knowledge, this study was the first PFMT mHealth app (interactive version) interventional pilot trial involving antenatal mothers at all parity at a government healthcare clinic. This study has a small sample size, almost similar to a previous PFMT app for nonpregnant women [52]. 

The limitation of this study was the pilot feasibility in its design, leading to a need to be interpreted with caution for its preliminary effectiveness outcomes due to having a small sample size, single centre, and short duration. Hence, this study was not powered to detect significant changes in adherence in PFMT. Other limitations were that the previous history of UI was not investigated, and the previous muscle tone was not recorded. Both these limitations were the confounder variables in this pilot RCT study. Therefore, in our future effectiveness RCT, these two factors will be assessed and included as the independent variables. Another limitation was that this study applied the sealed envelop for random allocation instead of block randomisation published in the protocol. However, this would not affect the preliminary effectiveness of this pilot RCT.

In the future, the app will be refined to improve the user interface, especially regarding knowledge acquisition and the training timer. The KEPT web will be further improved, to enable pregnant women to communicate with healthcare providers, especially when having doubts about PFMT and UI. Subsequently, for pregnant women with hypertonicity of their pelvic muscles, perhaps an additional interface to explain the other methods of Kegel exercise should be tailored to manage the ‘complicated group’.

## 5. Conclusions

This pilot study demonstrated the strategies that need to be implemented for the feasibility of our future RCT [53]. Additional incentives and eligibility screening at earlier trimesters (second trimester) may improve recruitment rates. Even though the preliminary effectiveness found significant improvement in knowledge, attitude, and self-efficacy, it did not improve PFMT adherence.

Therefore, this study added another line of evidence to our needs assessment studies [7,54], validation study [19], and formative research [26] in KEPT app development. The data demonstrated that pregnant women (with moderate-to-low income status) in our healthcare clinics are interested in the mHealth app intervention, indicating readiness for the realm of digital health.

## Figures and Tables

**Table 1:** Participant inclusion and exclusion criteria.

Inclusion Criteria	Exclusion Criteria
Malaysian citizen	Non-Malaysian citizen (due to Non-Malay speaking)
Mobile phone (Android) and internet access	Mobile phone (iPhone)
Pregnant woman	Planning to be pregnant or post-partum woman
Age more than 18 years	Age less than 18 years (Teenage pregnancy)
Any parity at 26–27 weeks gestation	Chronic medical problem(s) before pregnancy
Stress UI or Mixed UI (International Consultation on Incontinence Questionnaire-UI-Short Form [24,25])	Urge UI Complicated pregnancy (not advisable to perform PFMT)

**Table 2:** Preliminary effectiveness outcomes.

Outcome	Description
Primary outcome:	
PFMT Adherence	Increasing PFMT adherence from lowest score (0) to maximum score (24) of Exercise Adherence Rating Scale (EARS) [29].
Secondary outcomes:	
Urinary incontinence	Severity urinary incontinence symptoms using the International Consultation on Incontinence Questionnaire-Urinary Incontinence Short Form (ICIQ-UI SF) [24,25].
Quality of Life	To assess the quality of life among pregnant women with UI at baseline, one-month, and two-month post-intervention. International Consultation on Incontinence Questionnaire Urinary Incontinence-Lower Urinary Tract Symptom quality of life (ICIQ-LUTSqol) [24,25].
PFMT Knowledge, Attitude, and Practice	To assess the knowledge, attitude, and practices towards PFMT at baseline, one-month, and two-month post-intervention. Knowledge, attitude, and practice towards pelvic floor muscle training [30]
PFMT Self-efficacy	To measure the self-efficacy score at baseline, one-month, and two-month post-intervention. Self-Efficacy Scale For Practicing Pelvic Floor Exercise Questionnaire (SESPPFE) [31]

PFMT: pelvic floor muscle training.

**Table 3:** Baseline characteristics of the intervention vs. control group.

Characteristics	Overall (*n* = 26)	Baseline Comparison		Test	*p*-Value
		Intervention Group (*n* = 16)	Control Group (*n* = 10)		
Age (year), M ± SD	29.5 ± 4.8	29.7 ± 3.9	29.1 ± 6.2	*t*	0.772
BMI (kg m^2^), M ± SD	28.2 ± 3.9	28.7 ± 4.1	27.4 ± 3.7	*t*	0.428
Ethnicity, % (n)					
Malay	92.3 (24)	93.8 (15)	90.0 (9)		
Non-Malay	7.7 (2)	6.2 (1)	10.0 (1)	FET	1.0
Education, % (n)					
Primary and Secondary	30.8 (8)	31.3 (5)	30.0 (3)		
College/University	69.2 (18)	68.7 (11)	70.0 (7)	FET	1.0
Occupational, % (n)					
Unemployed	30.8 (8)	25.0 (4)	40.0 (4)		
Employed	69.2 (18)	75.0 (12)	60.0 (6)	FET	0.664
Parity, % (n)					
Nulliparous	34.6 (9)	37.5 (6)	30.0 (3)		
Multiparous ≥ 1	65.4 (17)	62.5 (10)	70.0 (7)	FET	1.0
Type of UI, % (n)					
SUI	57.7 (15)	62.5 (10)	50.0 (5)		
MUI	42.3 (11)	37.5 (6)	50.0 (5)	FET	0.689

FET: Fisher exact test, *t*-Test, *p* < 0.05 significance.

**Table 4:** Baseline outcome measures and comparison between intervention and control groups.

Outcome Measures	Overall (*n* = 26)	Baseline Comparison		Test	*p*-Value
		Intervention Group (*n* = 85)	Control Group (*n* = 85)		
UI Severity Score					
Median (IQR)	10.00 (5.0)	7.50 (4.0)	11.50 (6.0)	MWT	0.031 *
M ± SD	9.77 ± 5.76	7.88 ± 2.34	12.80 ± 8.15		
Quality of life					
Median (IQR)	30.50 (12.00)	29.50 (6.5)	36.50 (16.75)	MWT	0.135
M ± SD	32.85 ± 8.71	30.25 ± 5.95	37.00 ± 10.97		
PFMT Knowledge Score					
Median (IQR)	7.50 (6.75)	8.00 (8.25)	7.50 (6.0)	t	0.497
M ± SD	7.62 ±4.46	7.125 ± 4.43	8.40 ± 4.65		
PFMT Attitude Score					
Median (IQR)	31.00 (5.5)	31.0 (6.0)	31.5 (9.25)	MWT	0.391
M ± SD	29.01 ± 6.23	30.45 ± 5.20	30.60 ± 4.97		
PFMT Practice Score					
Median (IQR)	8.00 (5.00)	8.0 (5.0)	8.0 (4.5)	MWT	0.698
M ± SD	7.77 ± 2.82	7.94 ± 3.07	7.40 ± 2.50		
PFMT Self-Efficacy Score					
Median (IQR)	53.53 (43.68)	51.18 (47.94)	60.88 (42.65)	*t*	0.475
M ± SD	51.47± 26.21	48.49 ± 27.51	56.24 ± 24.62		
PFMT Adherence					
Median (IQR)	15.00 (4.50)	14.50 (6.8)	15.00 (3.00)	*t*	0.832
M ± SD	13.81 ± 4.23	13.25 ± 5.12	14.70 ± 2.50		

MWT: Mann–Whitney *U* Test, *t*-Test, * *p* < 0.05 significance.

**Table 5:** The effect of KEPT app on pelvic floor muscle training adherence.

Outcome Measures	β	SE	95%CI	*p*
PFMT Adherence				
Group effect ^a^	0.442	2.4276	−4.316 to 5.200	0.052
Time 2	2.369	1.0113	−4.442 to 5.126	0.889
Time 3	0.033	1.0113	0.387 to 4.351	0.019 *
Group*time 2 ^b^	1.154	2.7036	−4.145 to 6.453	0.670
Group*time 3 ^b^	−2.910	2.8799	−8.554 to 2.735	0.312

Data are presented as β: regression coefficient; SE: standard error; CI: confidence interval; Time 1–3 refer to baseline, 1 month and 2 months post-intervention, respectively. Reference: control group and baseline are the references for group effect and time 1–3, respectively. ^a^ Group effect: the difference between groups at 1 month and 2 months post-intervention; Time 2–3: the time effect on control group at 1 month and 2 months post-intervention, respectively, compared with baseline; ^b^ Group*time: the difference of the change between two groups at 1 month and 2 months post-intervention, respectively, compared with baseline. * *p* < 0.05 significance.

**Table 6:** The effect of KEPT app on PFMT knowledge, attitude, practice, and self-efficacy.

Outcome Measures	β	SE	95%CI	*p*
PFMT knowledge				
Group effect ^a^	−1.343	1.6716	−4.619 to 1.933	0.422
Time 2	−0.473	0.6801	−1.806 to 0.860	0.487
Time 3	9.218	5.5097	−1.5812 to 0.017	0.094
Group*time 2 ^b^	2.968	1.1630	0.688 to 5.247	0.011 *
Group*time 3 ^b^	−3.708	5.8277	−15.130 to 7.714	0.525
PFMT attitude				
Group effect ^a^	−2.642	2.2175	−6.988 to 1.704	0.233
Time 2	−3.552	2.8517	−9.141 to 2.037	0.213
Time 3	−0.309	1.9151	−4.062 to 3.445	0.872
Group*time 2 ^b^	6.246	4.0333	−1.659 to 4.151	0.121
Group*time 3 ^b^	5.884	2.7729	0.449 to 11.319	0.034 *
PFMT practice				
Group effect ^a^	0.530	1.0594	−1.547 to 2.606	0.617
Time 2	1.352	0.6657	0.048 to 2.657	0.042
Time 3	2.668	1.1254	0.463 to 4.874	0.018 *
Group*time 2 ^b^	0.924	1.1624	−1.355 to 3.202	0.427
Group*time 3 ^b^	0.179	1.6070	−2.971 to 3.329	0.911
PFMT Self-efficacy				
Group effect ^a^	−17.916	10.2310	−37.968 to 2.137	0.080
Time 2	−19.178	14.2831	−47.172 to 8.817	0.179
Time 3	−9.049	10.9787	−30.567 to 12.469	0.410
Group*time 2 ^b^	32.541	15.7129	1.745 to 63.338	0.038 *
Group*time 3 ^b^	16.939	14.4040	−11.293 to 45.170	0.240

Data are presented as β: regression coefficient; SE: standard error; CI: confidence interval; Time 1–3 refer to baseline, 1 month and 2 months post-intervention, respectively. Reference: control group and baseline are the references for group effect and time 1–3, respectively. ^a^ Group effect: the difference between groups at 1 month and 2 months post-intervention; Time 2–3: the time effect on control group at 1 month and 2 months post-intervention, respectively, compared with baseline; ^b^ Group*time: the difference of the change between two groups at 1 month and 2 months post-intervention, respectively, compared with baseline. * *p* < 0.05 significance.

**Table 7:** The effect of KEPT app on urinary incontinence and quality of life.

Outcome Measures	β	SE	95%CI	*p*
UI Symptom severity				
Group effect ^a^	−4.989	2.4942	−9.878 to −0.101	0.045 *
Time 2	−4.748	2.4159	−9.483 to −0.013	0.049 *
Time 3	−5.389	3.0569	−11.380 to 0.603	0.078
Group*time 2 ^b^	4.172	2.4941	−0.717 to 9.060	0.094
Group*time 3 ^b^	3.498	3.1421	−2.661 to 9.656	0.266
Quality of Life				
Group effect ^a^	−7.048	3.2752	−13.467 to −0.628	0.031 *
Time 2	0.000	0.0002	−0.001 to 0.000	0.328
Time 3	−2.000	2.2608	2.431 to 0.783	0.376
Group*time 2 ^b^	0	0.0002	0.000 to 0.001	0.253
Group*time 3 ^b^	−1.000	2.6965	−6.285 to 4.285	0.137

Data are presented as β: regression coefficient; SE: standard error; CI: confidence interval; Time 1–3 refer to baseline, 1 month and 2 months post-intervention, respectively. Reference: control group and baseline are the references for group effect and time 1–3, respectively. ^a^ Group effect: the difference between groups at 1 month and 2 months post-intervention; Time 2–3: the time effect on control group at 1 month and 2 months post-intervention, respectively, compared with baseline; ^b^ Group*time: the difference of the change between two groups at 1 month and 2 months post-intervention, respectively, compared with baseline. * *p* < 0.05 significance.
